# Explicit Motor Imagery Deficits in Individuals With Chronic Pain: A Systematic Review and Meta‐Analysis

**DOI:** 10.1155/prm/5805413

**Published:** 2026-09-07

**Authors:** Roy La Touche, Ferran Cuenca-Martínez, Jose Vicente León-Hernández, Alba Paris-Alemany, Miguel A. Sorrel, Álvaro Reina-Varona

**Affiliations:** ^1^ Programa de Doctorado Psicología, Facultad de Psicología, Universidad Autónoma de Madrid, Madrid, Spain, uam.es; ^2^ Departamento de Fisioterapia, Centro Superior de Estudios Universitarios La Salle, Universidad Autónoma de Madrid, Madrid, Spain, uam.es; ^3^ Motion in Brains Research Group, Centro Superior de Estudios Universitarios (CSEU) La Salle, Universidad Autónoma de Madrid, Madrid, Spain, uam.es; ^4^ Institute of Neurosciences and Craniofacial Pain (INDCRAN), Madrid, Spain; ^5^ Department of Physiotherapy, University of Valencia, C/Gascó Oliag, Valencia 46010, Spain, uv.es; ^6^ Department of Basic Health Sciences, Universidad Rey Juan Carlos, Alcorcón, Madrid, Spain, urjc.es; ^7^ Department of Social Psychology and Methodology, Faculty of Psychology, Autonomous University of Madrid, Madrid, Spain, uam.es

**Keywords:** chronic pain, mental ability impairments, mental chronometry, motor imagery, vividness

## Abstract

**Background:**

Chronic pain has been associated with disrupted sensorimotor integration and altered internal body representations. While implicit motor imagery (MI) has been explored in pain populations, explicit MI, defined as the deliberate mental simulation of movement, remains less well understood.

**Objective:**

This systematic review and meta‐analysis aimed to examine differences in explicit MI ability, vividness, and mental chronometry between individuals with chronic pain and healthy controls.

**Methods:**

We screened six databases for observational studies comparing explicit MI outcomes in chronic pain or clinically relevant persistent/recurrent pain‐related conditions versus asymptomatic healthy populations. Random‐effects and multilevel meta‐analyses were conducted using standardized mean differences (SMD) and 95% confidence and prediction intervals. Methodological quality was assessed with the JBI and NOS tools.

**Results:**

Twelve studies (*n* = 1603) met inclusion criteria. Individuals with pain‐related clinical conditions showed moderate‐to‐large deficits in MI ability (SMD = −0.96), vividness (SMD = 1.06 for kinesthetic vividness), and mental chronometry (SMD = 0.84), although heterogeneity varied across outcomes. Mental chronometry showed the most consistent pattern across studies, whereas imagery ability and vividness were associated with greater between‐study variability. Correlational analyses identified perceived disability, self‐efficacy, and kinesiophobia as relevant moderators.

**Discussion:**

Individuals with chronic pain appear to display significant impairments in explicit MI, particularly in temporal simulation and some kinesthetic dimensions. However, these findings should be interpreted in light of the clinical and methodological heterogeneity across included studies.

## 1. Introduction

Chronic pain is defined by its persistence or recurrence for more than 3 months [1]. This condition has been linked to neuroplastic changes in the central nervous system, affecting not only pain modulation but also sensorimotor integration and body representation [2–5]. For instance, in chronic low back pain, functional alterations have been observed in the organization of somatosensory cortices and higher‐order motor areas, alongside hyperactivation in regions involved in pain processing [6].

The sensorimotor theory of pathological pain suggests that a mismatch between motor intention and sensory feedback contributes to pain persistence, highlighting the need to investigate the mechanisms underlying movement planning and internal representation [7, 8]. In this context, motor imagery (MI) has been proposed as a valuable tool for exploring how chronic pain affects the ability to mentally simulate movement. MI refers to the ability to evoke a movement mentally without physically executing it [9, 10], engaging neural networks that largely overlap with those involved in actual movement execution [11].

The literature distinguishes two primary modalities of MI: implicit and explicit. Implicit MI involves the discrimination of motor attributes without conscious awareness of imagining movement and is commonly assessed using the left/right judgment task. While systematic reviews have examined this process in peripheral musculoskeletal conditions, the findings are less consistent in axial chronic pain [12–15]. Conversely, explicit MI entails the deliberate generation of a motor image, enabling modulation of its vividness and detail [9, 16]. This process primarily activates the premotor cortex, supplementary motor, and somatosensory areas [11, 17].

The available evidence on explicit MI in chronic pain remains more fragmented compared to that on implicit MI. Some studies suggest that neuropathic pain is associated with heightened cortical activation during imagery tasks, which may decrease following targeted interventions [18, 19]. Similarly, altered activation patterns have been observed in nociceptive regions when patients with chronic cervical pain imagine potentially painful actions [20]. These findings indicate that the conscious simulation of motor acts may be modulated by chronic pain, with potential consequences for sensorimotor cortical plasticity [3, 7]. Specifically, in cases of nonspecific chronic low back pain, patients tend to require more time both to imagine and to execute movements, exhibiting greater discrepancies between actual execution time and mental representation in functional tasks [21].

Despite these insights, no systematic reviews or meta‐analyses have specifically addressed explicit MI in chronic pain. Existing syntheses have predominantly focused on implicit MI or peripheral musculoskeletal conditions, leaving key questions unexamined regarding how chronic pain affects motor image generation, imagery vividness, and the temporal dynamics of imagery [12, 13]. Given the importance of motor intention–sensory feedback coherence, as proposed by the sensorimotor theory [7, 8], it is crucial to investigate explicit MI alterations across different chronic pain conditions and to identify factors contributing to variability in the reported findings.

This systematic review and meta‐analysis will specifically focus on explicit MI, assessing the ability to generate motor images, imagery vividness, and imagery chronometry in individuals with pain compared to asymptomatic controls. In addition, we will qualitatively examine psychological variables (e.g., anxiety, catastrophizing, and fear of movement) and sensory factors (e.g., pain type and sensory disturbances) that may be associated with MI performance. Through this approach, we aim to provide a comprehensive perspective on alterations in explicit MI and the underlying mechanisms contributing to pain persistence, ultimately informing future research and clinical interventions.

## 2. Methods

This systematic review was recorded in the International Prospective Register of Systematic Reviews (PROSPERO) under the registration number CRD420251003473. The review process adhered to the Preferred Reporting Items for Systematic Reviews and Meta‐Analyses (PRISMA) guidelines as outlined by [22].

### 2.1. Inclusion Criteria

#### 2.1.1. Population

Adults older than 18 years with chronic pain conditions were eligible for inclusion in the case group. Studies involving recurrent or persistent pain‐related clinical conditions were also considered when the sample reflected clinically relevant ongoing pain symptomatology. Comparator groups consisted of asymptomatic, pain‐free, healthy adults within the same age range. Both groups were required to meet the eligibility criteria regarding age and general health status.

To improve methodological clarity, the eligibility criteria were structured according to the PICO framework as follows: Population: adults older than 18 years with chronic pain or clinically relevant recurrent/persistent pain‐related conditions; Comparator: asymptomatic, pain‐free, healthy adults; Outcomes: explicit MI ability, vividness, and mental chronometry; Study design: observational comparative studies.

#### 2.1.2. Outcome Measures

Three primary outcome measures were assessed:

1. Ability to generate MI: This variable refers to how readily participants can form an internal mental representation of a movement. A common method of assessment is the Motor Imagery Questionnaire (MIQ), in which participants are first asked to perform a movement, then to imagine performing it—either through visual or kinesthetic modalities of MI—and subsequently rate the difficulty of generating the mental representation.
2. Vividness of VMI: This outcome describes how realistic or detailed the imagined movement appears, encompassing both visual and kinesthetic MI. Tools such as the VMI Questionnaire (VMIQ) assess different modalities (e.g., internal/external visual imagery and kinesthetic imagery).
3. Mental chronometry of MI: This outcome focuses on the time participants require to mentally simulate a movement, independently of actual execution. Only the duration of the imagined task is recorded, excluding any measurement of real movement performance.

#### 2.1.3. Study Design

Observational studies—including cross‐sectional, case‐control, and cohort designs—were eligible for inclusion, provided they met the above criteria for participants and outcomes.

### 2.2. Data Sources and Searches

Two independent reviewers conducted the literature search up to February 20th, 2025, using the following electronic databases: PubMed, Embase, EBSCO, Web of Science, PEDro, and Google Scholar. Differences that emerged during this phase were resolved by consensus. No filters regarding language, population, or study design were applied. The search strategy combined Medical Subjects’ Headings (MeSH [“Chronic Pain”]), [“Pain”], or [“Low Back Pain”], and non‐MeSH terms (“Motor Imagery”, “Mental Imagery Ability”, “Movement Imagery”, “Kinesthetic Imagery”, “Visual Imagery”, “Mental Practice”, or “Movement Representation”, among others) adding a Boolean operator (AND and/or OR) to combine them. Table 1 displays the search strategies employed, which were adapted for each database.

**TABLE 1:** Search and selection process: search engines, databases, search equations, and results.

Search engine	Databases	PICOS components		Equation	Date	Registries (*n*)
		Population	Outcome			
PubMed	MEDLINE	Patients with chronic pain	MI	(“Chronic Pain”[Title/Abstract] OR “Chronic Pain”[MeSH Terms] OR “Neuropathies”[Title/Abstract] OR “Neuralgia”[Title/Abstract] OR “Neuralgia”[MeSH Terms] OR “chronic primary pain”[Title/Abstract] OR “widespread chronic pain”[Title/Abstract] OR “persistent pain”[Title/Abstract] OR “neuropathic pain”[Title/Abstract] OR “nociplastic pain”[Title/Abstract] OR “Chronic Low Back Pain”[Title/Abstract] OR “CLBP”[Title/Abstract] OR “Cervical Pain”[Title/Abstract] OR “Chronic Neck Pain”[Title/Abstract] OR “Chronic Shoulder Pain”[Title/Abstract] OR “Low Back Pain”[Title/Abstract] OR “Chronic Cervical Pain”[Title/Abstract] OR “Headache”[Title/Abstract] OR “Facial Pain”[Title/Abstract]) AND (“Motor Imagery”[Title/Abstract] OR “Motor Imagery Ability”[Title/Abstract] OR “Mental Imagery”[Title/Abstract] OR “Mental Imagery Ability”[Title/Abstract] OR “Motor Imagery Capacity”[Title/Abstract] OR “Skill Imagery”[Title/Abstract] OR “Movement Imagery”[Title/Abstract] OR “Movement Imagery Ability”[Title/Abstract] OR “Kinesthetic Imagery”[Title/Abstract] OR “Kinesthetic Imagery Ability”[Title/Abstract] OR “Visual Imagery”[Title/Abstract] OR “Visual Imagery Ability”[Title/Abstract] OR “Movement Imagination”[Title/Abstract] OR “Action Imagination”[Title/Abstract] OR “Mental Practice”[Title/Abstract] OR “Mental Practice Ability”[Title/Abstract] OR “Movement Representation”[Title/Abstract] OR “Motor Representation”[Title/Abstract])	26^th^ February, 2025	206
Embase	—	Patients with chronic pain	MI	(‘chronic pain’/exp OR ‘chronic intractable pain’ OR ‘chronic pain’ OR ‘chronic persistent pain’ OR ‘pain, chronic’ OR ‘spinal pain’/exp OR ‘pain, spinal’ OR ‘rachialgia’ OR ‘spinal pain’ OR ‘vertebral pain’ OR ‘low back pain’/exp OR ‘acute low back pain’ OR ‘back pain, low’ OR ‘chronic low back pain’ OR ‘loin pain’ OR ‘low back pain’ OR ‘low backache’ OR ‘low backpain’ OR ‘lowback pain’ OR ‘lower back pain’ OR ‘lumbago’ OR ‘lumbal pain’ OR ‘lumbal syndrome’ OR ‘lumbalgesia’ OR ‘lumbalgia’ OR ‘lumbar pain’ OR ‘lumbar spine syndrome’ OR ‘lumbodynia’ OR ‘lumbosacral pain’ OR ‘lumbosacral root syndrome’ OR ‘lumbosacroiliac strain’ OR ‘pain, low back’ OR ‘pain, lumbosacral’ OR ‘strain, lumbosacroiliac’ OR ‘chronic neck pain’/exp OR ‘chronic cervical pain’/exp OR ‘chronic shoulder pain’/exp OR ‘neuropathic pain’/exp OR ‘neuro‐pathic pain’ OR ‘neurogenic pain’ OR ‘neuropathic pain’ OR ‘pain, neuropathic’ OR ‘neuralgia’/exp OR ‘alveolar neuralgia’ OR ‘cardiac neuralgia’ OR ‘cervico occipital neuralgia’ OR ‘degenerative neuralgia’ OR ‘epileptiform neuralgia’ OR ‘hallucinatory neuralgia’ OR ‘idiopathic neuralgia’ OR ‘mammary neuralgia’ OR ‘mandibular joint neuralgia’ OR ‘nasociliary neuralgia’ OR ‘nerve pain’ OR ‘neuralgia’ OR ‘neuralgia facialis vera’ OR ‘neuralgia, rheumatic’ OR ‘neuralgic pain’ OR ‘neuralgy’ OR ‘neurodynia’ OR ‘neurologic pain’ OR ‘neurological pain’ OR ‘occipital neuralgia’ OR ‘peripheral neuralgia’ OR ‘pharyngeal neuralgia’ OR ‘red neuralgia’ OR ‘reminiscent neuralgia’ OR ‘rheumatic neuralgia’ OR ‘segmental neuralgia’ OR ‘sphenopalatine neuralgia’ OR ‘supraorbital neuralgia’ OR ‘symptomatic neuralgia’ OR ‘trifacial neuralgia’ OR ‘vidian neuralgia’ OR ‘visceral neuralgia’) AND (‘motor imagery’/exp OR ‘explicit motor imagery’ OR ‘motor imagery ability’ OR ‘mental imagery’/exp OR ‘mental imagery ability’ OR ‘movement imagery questionnaire’/exp OR ‘movement imagery’ OR ‘mental simulation’/exp OR ‘mental rehearsal’ OR ‘mental simulation’ OR ‘mental visualization’ OR ‘mental visualization’ OR ‘kinesthetic motor imagery’/exp OR ‘visual motor imagery’)	26^th^ February, 2025	51
EBSCO	Academic Search Premier; Education Source; ERIC; Library, Information Science & Technology Abstracts; MEDLINE Complete; OpenDissertations; PSICODOC; Sociology Source Ultimate; Teacher Reference Center; The Serials Directory	Patients with chronic pain	MI	(motor imagery or mental imagery or visualization or mental rehearsal or mental practice) AND (chronic pain or persistent pain or long‐term pain or long‐term pain) AND (observational studies: cohort and case‐control studies)	26^th^ February, 2025	80
Web of Science	Web of Science Core Collection; Current Contents Connect; Derwent Innovations Index; KCI‐Korean Journal Database; MEDLINE; ProQuest Dissertations & Theses Citation Index; SciELO Citation Index	Patients with chronic pain	MI	AB = (“Chronic Pain”) AND AB = (“motor imagery”)	26^th^ February, 2025	356
PEDro	—	Patients with chronic pain	MI	“motor imagery” AND “Chronic Pain”	26^th^ February, 2025	13

### 2.3. Selection Process

Titles, abstracts, and full‐text articles identified through the initial search were independently screened by two reviewers using the Rayyan AI tool. If the information provided in a study was insufficient to determine eligibility, the corresponding authors were contacted for clarification. Studies categorized as non–peer‐reviewed publications, news items, popular science articles, study protocols, or those lacking full‐text availability were excluded. Any disagreements during the final stage of inclusion were resolved through discussion with a third reviewer.

### 2.4. Data Extraction

Initially, the two independent reviewers screened the studies to assess their relevance of the research questions and objectives. The first screening was based on the title, abstract, and keywords of each study. If there was no consensus or if the abstract provided insufficient information, the full text was reviewed. In the second phase of the screening, full texts were assessed to confirm whether the studies met all inclusion criteria. Discrepancies between reviewers were resolved through discussion and consensus, with mediation by a third reviewer when necessary. Data extraction was carefully structured to ensure the comprehensive retrieval of all relevant information for the Results section.

### 2.5. Methodological Quality Assessment

The methodological quality of the included cross‐sectional studies was evaluated using two tools:

1. ‐Newcastle–Ottawa Scale (NOS) was adapted for cross‐sectional studies [23].
  1. This scale consists of seven items across three domains (Selection, Comparability, and Outcome), with a total possible score ranging from 0 to 10 stars. Its interrater reliability has been reported as moderate [24]. Following the criteria proposed by Elizagaray‐Garcia et al. [25], the final classification of each study was determined as follows: (a) High quality: 3 or 4 stars in selection, 1 or 2 in comparability, and 2 or 3 in outcome. (b) Moderate quality: 2 stars in selection, 1 or 2 in comparability, and 2 or 3 in outcome. (c) Low quality: 0 or 1 star in selection, 0 in comparability, and 0 or 1 in outcome.
2. Joanna Briggs Institute (JBI) Critical Appraisal Checklist for analytical cross‐sectional studies [26].
   This checklist includes 8 items, each with four possible responses (“Yes,” “No,” “Unclear,” and “Not applicable”). A “Yes” response earns 1 point, while the other three options receive 0 points, resulting in a total possible score of 0–8. Higher scores reflect better methodological quality.

Both tools were independently applied by two reviewers to each included study. Interrater agreement was assessed using Cohen’s kappa coefficient and interpreted according to the following classification: almost perfect (*κ* = 0.81–1.00), substantial (*κ* = 0.61–0.80), moderate (*κ* = 0.41–0.60), fair (*κ* = 0.21–0.40), slight (*κ* = 0.00–0.20), or poor (*κ* < 0.00) [27]. Any discrepancies were resolved by consensus, and if necessary, a third reviewer was consulted to reach a final decision.

### 2.6. Evidence Synthesis

The framework for evidence synthesis in this review was adapted from the approach described by van Tulder et al. [28] and further refined by La Touche et al. [29]. The classification of levels of evidence was defined according to the following criteria:

• No evidence: No observational studies (e.g., cross‐sectional or longitudinal) are available on the topic.
• Contradictory evidence: The findings from multiple observational studies (cross‐sectional or longitudinal) are inconsistent.
• Limited evidence: Available data are drawn from a single low‐quality case–control or cohort study and/or from at least two low‐quality cross‐sectional studies. In this review, the category was broadened to include one or two low‐quality and/or one or two moderate‐quality cross‐sectional studies.
• Moderate evidence: Consistent findings reported in multiple low‐quality case–control, cohort, or cross‐sectional studies, or from a single high‐quality case–control or cohort study. In this review, this category was extended to include one or two high‐quality cross‐sectional studies.
• Strong evidence: Consistent findings from at least three high‐quality case–control, cohort, or cross‐sectional studies.

### 2.7. Statistical Analysis

A series of random‐effects meta‐analyses were conducted using the metafor package (https://cran.r-project.org/web/packages/metafor/index.html) in R (R Core Team, 2022) in RStudio (Version 2023.06.0‐431) [30], based on the guideline “Doing Meta‐analysis with R: A Hands‐On Guide” [31]. Standardized mean differences (SMD) and associated variances were first computed via the escalc() function for each comparison between individuals with pain and healthy controls. To ensure interpretative consistency across pooled outcomes, effect sizes were calculated as the SMD between the pain group and the healthy control group, and the direction of each effect was explicitly oriented so that it consistently reflected poorer explicit MI performance in the pain group. For imagery ability outcomes (e.g., MIQ‐R and MIQ‐3), higher scores reflect better performance; therefore, negative SMDs indicated poorer performance in the pain group. For imagery vividness outcomes (e.g., VMIQ, VMIQ‐2, and TMIQ), scales were aligned so that the group with poorer vividness consistently showed the corresponding effect direction, with sign inversion applied when necessary (e.g., for the TMIQ, where higher scores indicate better vividness). For mental chronometry outcomes, higher values indicate slower and therefore poorer performance, so that positive SMDs indicated poorer performance in the pain group. In all cases, the direction of each effect size was verified to ensure that the pooled estimate consistently reflected poorer explicit MI performance in the pain group relative to controls.

We had to estimate the means and standard deviations from the median and quartiles in two studies [32, 33], using the 14th and 15th formulas of Wan et al. [34]. For each analysis, a restricted maximum‐likelihood (REML) estimator was used [35], with either a univariate random‐effects model or a multilevel random‐effects model when multiple effect sizes were nested within the same study that shares the same control [33]. In these multilevel models, a random intercept was specified at both the study and effect‐size levels to account for within‐study correlations [36]. A t‐distribution was considered for each meta‐analysis, applying the Knapp–Hartung adjustment for the univariate meta‐analyses and adjusting the degrees of freedom for the t‐distribution of the multilevel with the dfs = “contain” argument of the rma.mv function [37]. All analyses used a two‐sided significance threshold (*α* = 0.05) with a 95% confidence interval (95% CI).

Heterogeneity was assessed using Cochran’s *Q* statistic with corresponding *p* value, its associated *I* ^2^ statistic, representing the proportion of total variance attributable to between‐study heterogeneity, and the Tau value (*τ*) [38]. For multilevel models, the var.comp() function was employed to quantify the variance components at each hierarchical level and to compute total *I* ^2^. Additionally, 95% prediction intervals (95% PI) were estimated to describe the plausible range of true effects in future studies [39].

Forest plots were generated for each analysis to display study‐level effect sizes, corresponding to 95% CI, and model‐derived summaries (including weights and 95% PI). Funnel plots were used to visually inspect the potential presence of publication bias or small‐study effects, with confidence region shading set at 90%, 95%, and 99% [40]. When relevant, single‐study influence analyses and sensitivity analyses (e.g., removing outlying or influential cases and refitting the models) were carried out to evaluate the robustness of results; this included leave‐one‐out analyses via leave1out() or omitting specific studies that were suspected to drive heterogeneity [39].

## 3. Results

### 3.1. Study Selection Process

**FIGURE 1:**
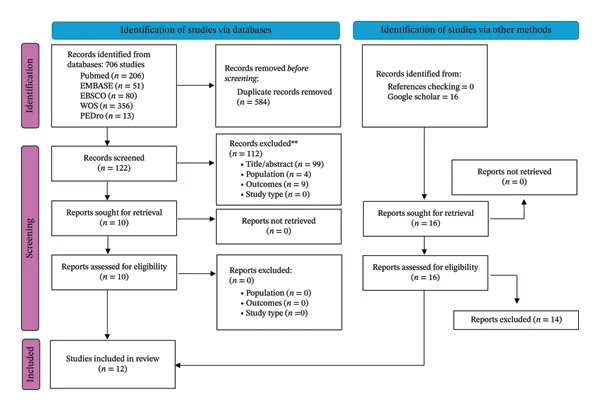
PRISMA flowchart of studies selection.

A total of 12 observational studies—11 cross‐sectional and 1 cohort study—were included in this systematic review [21, 32, 33, 41–49]. The study selection process is summarized in the revised PRISMA 2020 flow diagram shown in Figure 1, which was checked to ensure full numerical consistency across identification, screening, eligibility, and inclusion stages. Table 2 presents the characteristics of the included studies (study design, participants, inclusion/exclusion criteria, outcomes, instruments, procedure, main results, and main conclusions).

**TABLE 2:** Characteristics of the included studies.

Authors	Study design	Participants (*n*, age, diagnosis)	Inclusion/exclusion criteria	Variables of interest	Instruments/tests used	Conditions/procedure	Main results	Main conclusions
Ozcan & Karaali Kul (2022)	Cross‐sectional	Cases (CNPG): *n* = 83, 18–24 years, chronic neck pain; Controls (CG): *n* = 91, 18–24 years, healthy	Inclusion: Young adults, chronic neck pain ≥ 3 months. Exclusion: surgery, neurological disorders, recent medication	Motor imagery (visual/kinesthetic), cervical pain intensity, disability, kinesiophobia	MIQ‐3, SF‐MPQ, NDI, TSK	Single‐session administration of MI, pain, and disability questionnaires	Lower kinesthetic and internal visual imagery in neck pain group; associated with disability and kinesiophobia	Chronic neck pain alters motor imagery (internal and kinesthetic); important to address kinesiophobia and disability
Scandola et al. (2022)	Cross‐sectional	FM Group: *n* = 30 women, avg. age ∼48; Control: *n* = 30 women, avg. age ∼50	Inclusion: Women confirmed FM diagnosis. Exclusion: men, neurological conditions, severe comorbidities	Body perception/illusions, motor imagery, clinical features of FM	BoFI, VMIQ‐2, WPI, SSS, FIQ, HADS	Hospital‐based interview and questionnaires assessing body perception and MI	FM group showed frequent body illusions and lower motor imagery, correlated with reduced functionality	FM involves alterations in body representation and motor imagery; specific interventions necessary
Estradera‐Bel et al. (2024)	Cross‐sectional	NSCLBP group: *n* = 28, 18–65 years, chronic low back pain; Control: *n* = 28, 18–65 years, healthy	Inclusion: Adults, chronic low back pain ≥ 3 months. Exclusion: neurological or severe psychiatric conditions	Temporal congruence (MI vs. execution), disability, kinesiophobia, self‐efficacy, physical activity	MIQ‐R, TSK‐11, RMDQ, CPSS, IPAQ‐S	Temporal congruence assessment (MI vs. execution), questionnaires on disability and fear of movement	Temporal incongruence in MI observed in chronic low back pain, associated with disability and fear	Chronic low back pain shows MI‐execution dissociation; addressing cognitive/emotional factors is essential
Díaz‐Sáez et al. (2020)	Cohort study	CLBP patients: *n* = 30, 18–65 years; Controls: *n* = 30, 18–65 years	Inclusion: Adults, chronic low back pain ≥ 3 months. Exclusion: previous lumbar surgery, neurological or psychiatric disorders	Autonomic nervous system activity (MI vs. AO), kinesiophobia, catastrophizing, disability, self‐efficacy	MIQ‐R, TSK, PCS, RMDQ, CPSS, Biofeedback	Comparison of autonomic response in MI vs. action observation (AO), psychosocial questionnaires	Higher autonomic activation in chronic back pain patients (especially high kinesiophobia) during MI/AO	MI and AO trigger similar autonomic responses; kinesiophobia significantly influences chronic back pain responses
Alikaj et al. (2024)	Cross‐sectional	FMF group: *n* = 30, 18–65 years, FMF diagnosis; Control: *n* = 30, 18–65 years, healthy volunteers	Inclusion: Confirmed FMF diagnosis. Exclusion: neurological or severe rheumatic diseases, cancer	Motor imagery capacity, pain characteristics, pain catastrophizing	MIQ‐3, MPQ, PCS	Evaluation of MI ability, pain, and catastrophizing through questionnaires	FMF patients showed poorer motor imagery; catastrophizing negatively correlated with internal visual imagery	FMF linked to reduced motor imagery; catastrophizing especially affects internal visual imagery
Grande‐Alonso et al. (2025)	Cross‐sectional	CSP patients: *n* = 15, 18–65 years, unilateral shoulder pain; Controls: *n* = 15, 18–65 years, healthy	Inclusion: Adults, unilateral shoulder pain ≥ 3 months. Exclusion: previous surgery, radiculopathy, severe systemic diseases	MI capacity, pain intensity, disability, psychosocial factors, motor factors (ROM, grip strength)	MIQ‐R, Quick‐DASH, HADS, TSK‐11, PCS, CPSS	Evaluation of MI capacity, muscle strength, ROM, and psychosocial factors via questionnaires	Chronic shoulder pain showed reduced visual/kinesthetic imagery and increased mental timing; positively correlated with self‐efficacy and strength	Shoulder pain reduces imagery capacity; psychosocial and motor factors relevant for rehabilitation
Matesanz‐García et al. (2023)	Cross‐sectional	CTS patients: *n* = 20, ≥ 1 year symptoms; Controls: *n* = 18, similar age/sex	Inclusion: Confirmed CTS, symptoms ≥ 1 year. Exclusion: other neuropathies, previous surgery, fractures	MI capacity, imagined task timing, pain, neuropathic features, emotional state, pain processing	MIQ‐R, Boston Carpal, DN4, VAS, BDI, STAI, TSK	Cross‐sectional evaluation of MI and mental timing, neuropathic pain, and sensory modulation mechanisms	CTS patients showed greater visual imagery difficulty, longer latency; linked to central sensitization	Neuropathic pain in CTS affects motor imagery, linked to functional deficit and central sensitization phenomena
Alaca et al. (2025)	Cross‐sectional	*n* = 93 (48 rotator cuff‐related shoulder pain, 45 healthy controls); Avg. age: 46 years (pain group), 44 years (controls)	Inclusion: Adults, rotator cuff‐related shoulder pain ≥ 6 months. Exclusion: bilateral pain, previous surgery, systemic conditions	Tactile acuity, left/right discrimination, motor imagery, pressure pain threshold, central sensitization	TPDT, LRJT, VMIQ‐2, PPT, SPADI, FABQ, PCS, CSI	Bilateral assessment of tactile perception, MI, pain sensitivity, and psychosocial factors	Shoulder pain showed poorer motor imagery, higher tactile threshold, lower pain threshold; worse in central sensitization	Perceptual alterations and reduced imagery in shoulder pain; central sensitization important for clinical management
Cohen‐Aknine et al. (2025)	Cross‐sectional	*n* = 123 (CRPS = 40, CLP = 40, healthy = 43); median ages: 54, 49, 47, respectively	Inclusion: Adults with CRPS and CLP ≥ 3 months. Exclusion: amputation, fibromyalgia, pregnancy, psychiatric conditions	Explicit visual/kinesthetic MI	MIQ‐RS	Explicit MI assessment on both sides of the body	No differences in explicit imagery between groups or sides; Bayesian analysis supports no differences	Explicit motor imagery preserved in CRPS/CLP; recommended for chronic pain therapy
La Touche et al. (2019)	Cross‐sectional	*n* = 200 (100 chronic low back pain, 100 controls); Age: 18–65 years	Inclusion: Adults, chronic low back pain ≥ 3 months. Exclusion: previous spinal surgery, neurological conditions	Visual/kinesthetic motor imagery, timing, psychological factors	MIQ‐R, TSK‐11, PCS, CPSS, RMDQ	Assessment of visual/kinesthetic MI, timing, psychological factors, and disability	Significant MI difficulty in chronic low back pain; associated with disability and fear	Chronic low back pain impairs motor imagery; psychological factors key for rehabilitation

*Note:* TSK: Tampa Scale for Kinesiophobia; FM: fibromyalgia; BoFI: body feelings and illusions; VMIQ‐2: Vividness of Movement Imagery Questionnaire‐2; HADS: Hospital Anxiety and Depression Scale; CPSS: Chronic Pain Self‐Efficacy Scale; Quick‐DASH: Disabilities of the Arm, Shoulder, and Hand Questionnaire; MIQ‐RS: Movement Imagery Questionnaire‐Revised Second Edition; BodyTAP: Trinity Assessment of Body Plasticity; TMD: Temporomandibular Disorders; TMIQ: Tongue and Mouth Imagery Questionnaire; KVIQ‐10: Kinesthetic and Visual Imagery Questionnaire‐10 items.

Abbreviations: AO, action observation; BDI, Beck Depression Inventory; BFI, big five inventory; CLP, chronic limb pain; CRPS, complex regional pain syndrome; CSI, Central Sensitization Inventory; CTS, Carpal Tunnel Syndrome; DN4, Douleur Neuropathique 4; FABQ, Fear Avoidance Beliefs Questionnaire; FIQ, Fibromyalgia Impact Questionnaire; FMF, Familiar Mediterranean Fever; IPAQ‐S, International Physical Activity Questionnaire‐Short; LRJT, Left/Right Judgment Task; MI, motor imagery; MIQ‐3, Movement Imagery Questionnaire‐3; MIQ‐R, Movement Imagery Questionnaire Revised; MPQ, McGill Pain Questionnaire; NDI, Neck Disability Index; PCS, Pain Catastrophizing Scale; PPT, pressure pain threshold; RMDQ, Roland–Morris Disability Questionnaire; ROM, range of movement; SF‐MPQ, Short‐Form McGill Pain Questionnaire; SPADI, Shoulder Pain and Disability Index; SSS, Symptom Severity Scale; STAI, State‐Trait Anxiety Inventory; TAS, Tellegen Absorption Scale; TPDT, two‐point discrimination test; VAS, visual analog scale; WPI, Widespread Pain Index.

### 3.2. Methodological Quality Assessment

Overall, the included studies demonstrated moderate to good methodological quality. According to the JBI Critical Appraisal Checklist, 9 out of 12 studies scored ≥ 6 out of 8 [21, 41, 43–45, 47–49]. Based on the adapted NOS scale, 10 studies received ≥ 7 out of 9 stars, with the highest scores observed in the studies of La Touche et al. [45], Díaz‐Sáez et al. [43], and Alaca et al. [47]. The combined quality rating (JBI + NOS) classified seven studies as high methodological quality [21, 43–45, 47, 48] and five studies as moderate quality [32, 33, 41, 42, 46]. Interrater agreement was classified as almost perfect (*κ* = 0.86). The final combined result of the methodological quality is presented in Table 3, and detailed results for each appraisal tool are provided in Supporting Information 1 and 2.

**TABLE 3:** Combined methodological quality assessment (NOS and JBI).

Study	NOS (score/quality)	JBI (score)
Scandola et al. [41]	7/10—Moderate	7/8
La Touche et al. [45]	8/10—Good	7/8
Díaz‐Sáez et al. [43]	8/10—Good	7/8
Alvarado et al. [48]	7/10—Good	7/8
Ozcan & Karaali [32]	6/10—Moderate	5/8
Scandola et al. [42]	7/10—Moderate	6/8
Matesanz‐García et al. [44]	7/10—Moderate	7/8
Cohen‐Aknine et al. [33]	6/10—Moderate	5/8
Alikaj et al. [46]	7/10—Good	6/8
Estredera‐Bel et al. [21]	7/10—Good	7/8
Grande‐Alonso et al. [49]	7/10—Good	7/8
Alaca et al. [47]	8/10—Good	7/8

Abbreviations: JBI, Joanna–Briggs Institute; NOS, Newcastle–Ottawa Scale.

### 3.3. Data Extraction

This systematic review included a total of 1603 participants, comprising 808 individuals with chronic musculoskeletal pain and 795 asymptomatic controls. Participants’ ages ranged from 18 to 85 years, with a mean age of 43.9 years (SD = 13.1). Gender distribution varied across studies but was overall balanced.

The clinical conditions assessed encompassed a range of pain syndromes, including chronic low back pain, *n* = 3 studies [21, 43, 45]; shoulder pain, *n* = 2 studies [47, 49]; temporomandibular disorders, *n* = 1 study [48]; fibromyalgia, *n* = 1 study [42]; complex regional pain syndrome, *n* = 1 study [33]; lower limb pain, *n* = 1 study [33]; neck pain, *n* = 1 study [32]; familial Mediterranean fever, *n* = 1 study [46]; carpal tunnel syndrome, *n* = 1 study [44]; and spinal cord injury, *n* = 1 study [41].

### 3.4. Questionnaires Used to Assess Explicit MI

All 12 included studies focused on the assessment of explicit MI using validated self‐report instruments and behavioral tasks. The tools were classified according to the specific outcome assessed: imagery ability, imagery vividness, and mental chronometry.

• Imagery ability was evaluated in 8 studies using the *Movement Imagery Questionnaire-Revised* (MIQ‐R) or its updated version *MIQ-3* [21, 32, 33, 43–46, 49]. These instruments assess both kinesthetic and visual components of MI ability.
• Imagery vividness was assessed in 4 studies using the *Vividness of Movement Imagery Questionnaire* (VMIQ), either in its original or second version (VMIQ‐2), or the *Tongue and Mouth Imagery Questionnaire* (TMIQ). Specifically, VMIQ or VMIQ‐2 was applied in 3 studies [41, 42, 47], while the TMIQ was used in [48].
• Mental chronometry was examined in five studies, which assessed the temporal characteristics of MI (mental chronometry) by measuring the duration of imagined movements without physical execution. These studies used customized chronometric imagery tasks [21, 43–45, 49].

### 3.5. Meta‐Analysis Results by MI Outcomes

#### 3.5.1. MI Ability

**FIGURE 2:**
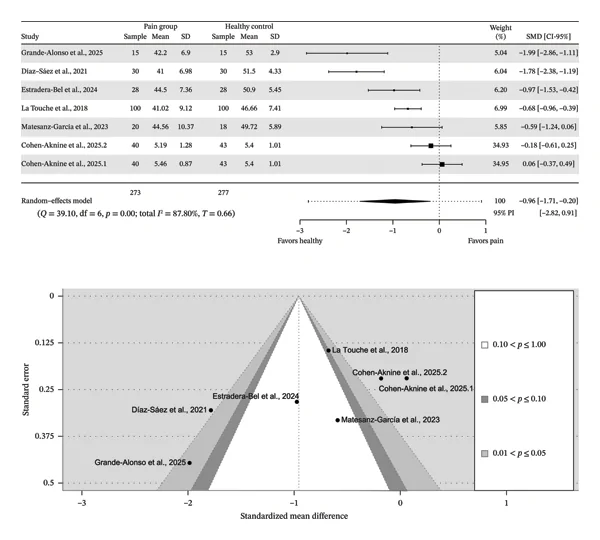
Synthesis forest and funnel plots of general motor imagery ability. The forest plot summarizes the results of the included studies (sample size, standardized mean differences [SMDs], and weight). The small boxes with the squares represent the point estimate of the effect size and sample size. The lines on either side of the box represent a 95% confidence interval (CI).

**FIGURE 3:**
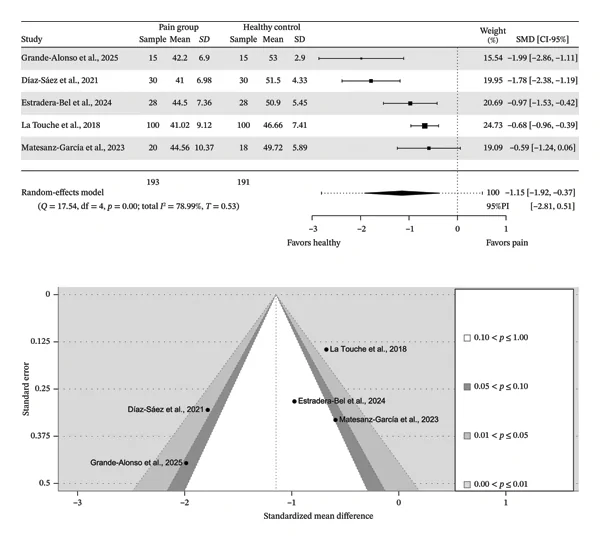
Exclusion sensitivity plot for general motor imagery ability.

The meta‐analysis included six studies assessing general MI ability in individuals with chronic musculoskeletal pain. The pooled analysis revealed a moderate‐to‐large effect size (SMD = −0.96; 95% CI: [−1.71, −0.20]), with high heterogeneity (*I* ^2^ = 87.80%) and a wide PI [−2.82, 0.91] (Figure 2). Sensitivity analyses confirmed the robustness of the results as exclusion of individual studies did not substantially alter the results. For example, removal of the study by Cohen‐Akine et al. [33] increased the effect size (SMD = −1.15; 95% CI: [−1.92, −0.37]) though heterogeneity remained high (*I* ^2^ = 78.99%) (Figure 3). Visual inspection of funnel plots did not indicate substantial asymmetry.

**FIGURE 4:**
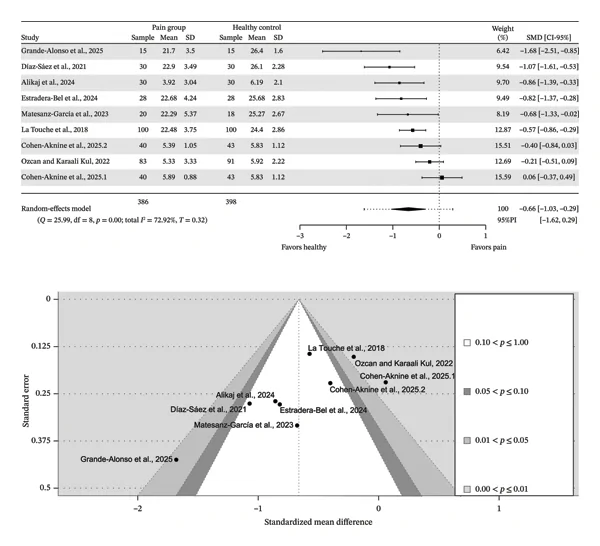
Synthesis forest and funnel plots of external visual motor imagery ability. The forest plot summarizes the results of the included studies (sample size, standardized mean differences [SMDs], and weight). The small boxes with the squares represent the point estimate of the effect size and sample size. The lines on either side of the box represent a 95% confidence interval (CI).

**FIGURE 5:**
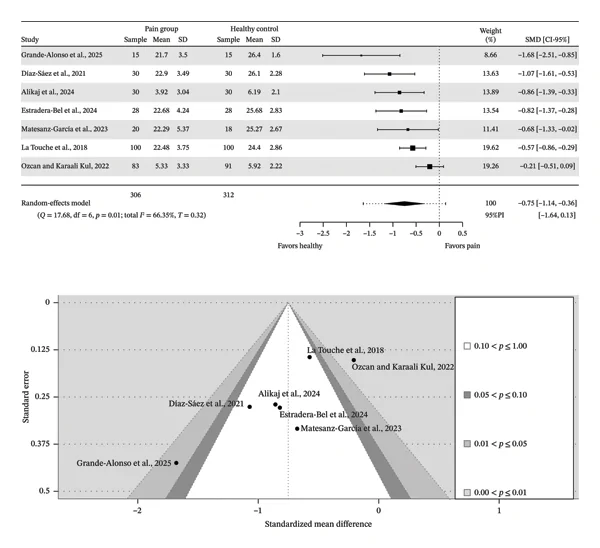
Exclusion sensitivity plot for external visual motor imagery ability.

Subscale analyses showed consistent patterns. External visual MI ability, evaluated in eight studies, showed a moderate effect size (SMD = −0.66; 95% CI: [−1.03, −0.29]), with high heterogeneity (*I* ^2^ = 72.92%) and a wide PI [−1.62, 0.29] (Figure 4). Sensitivity analyses confirmed the robustness of these findings. For instance, excluding Cohen‐Aknine et al. [33] increased the effect size (SMD = −0.75; 95% CI: [−1.14, −0.36]) with considerable heterogeneity (*I* ^2^ = 66.35%) (Figure 5). No substantial asymmetry was observed in funnel plots.

**FIGURE 6:**
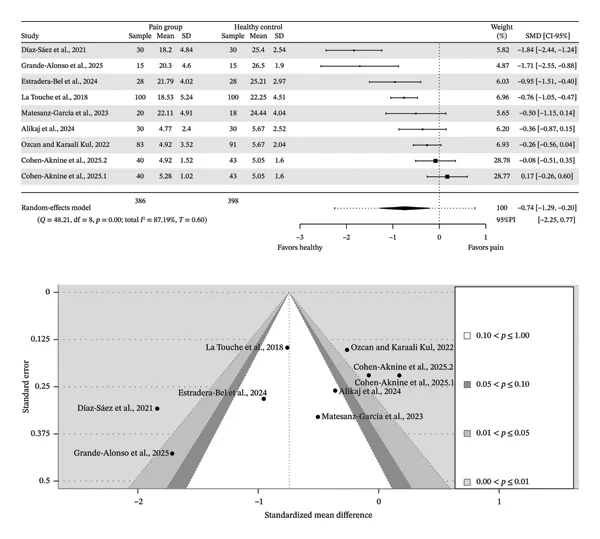
Synthesis forest and funnel plots of external kinesthetic motor imagery ability. The forest plot summarizes the results of the included studies (sample size, standardized mean differences [SMDs], and weight). The small boxes with the squares represent the point estimate of the effect size and sample size. The lines on either side of the box represent a 95% confidence interval (CI).

**FIGURE 7:**
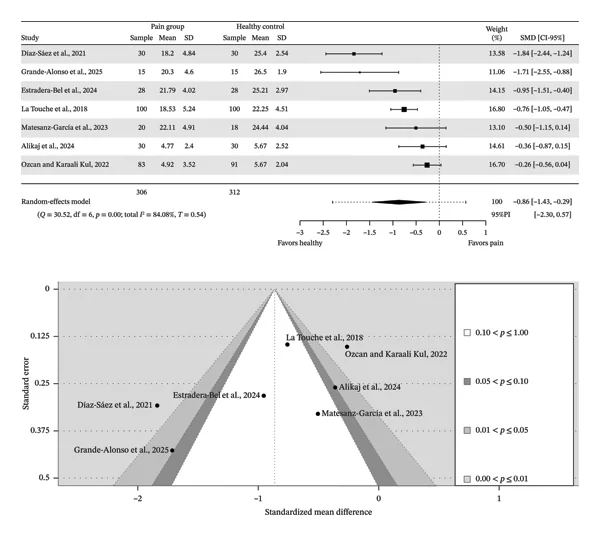
Exclusion sensitivity plot for external kinesthetic motor imagery ability.

Kinesthetic MI ability, assessed in eight studies, yielded a moderate effect size (SMD = −0.74; 95% CI: [−1.29, −0.20]) with high heterogeneity (*I* ^2^ = 87.19%) and a PI that included the null effect (PI: [−2.25, 0.77]) (Figure 6). Sensitivity analyses again confirmed the robustness of the results. Exclusion of Cohen‐Aknine et al. [33] resulted in an increased effect size (SMD = −0.86; 95% CI: [−1.43, −0.29]) with considerable heterogeneity (*I* ^2^ = 84.08%) (Figure 7). No significant asymmetry was observed in funnel plots.

##### 3.5.1.1. Evidence Synthesis

The findings, drawn from multiple studies of moderate to high methodological quality (seven studies with combined JBI/NOS scores ≥ 7/8), support a classification of moderate evidence. Notably, this level of evidence suggests that patients experiencing chronic musculoskeletal pain exhibit reduced motor imagery ability compared to healthy controls.

#### 3.5.2. MI Vividness

**FIGURE 8:**
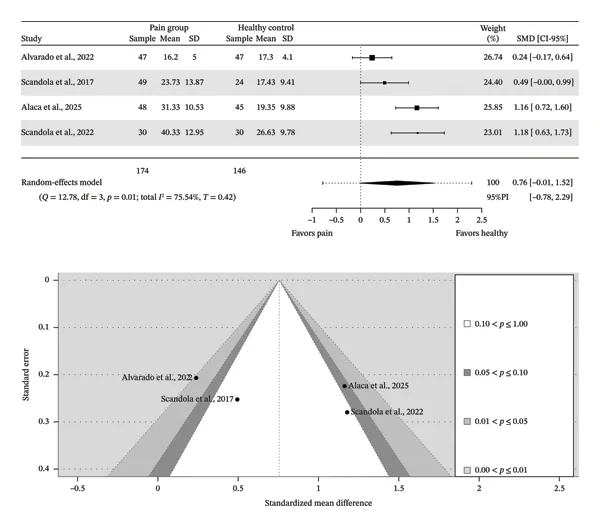
Synthesis forest and funnel plots of visual motor imagery vividness. The forest plot summarizes the results of the included studies (sample size, standardized mean differences [SMDs], and weight). The small boxes with the squares represent the point estimate of the effect size and sample size. The lines on either side of the box represent a 95% confidence interval (CI).

**FIGURE 9:**
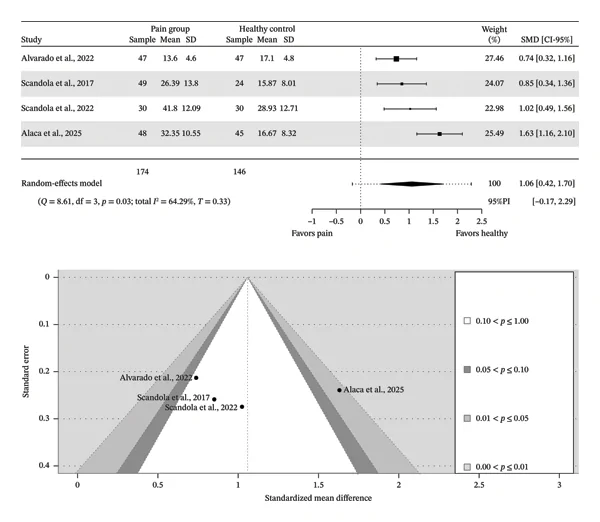
Synthesis forest and funnel plots of kinesthetic motor imagery vividness. The forest plot summarizes the results of the included studies (sample size, standardized mean differences [SMDs], and weight). The small boxes with the squares represent the point estimate of the effect size and sample size. The lines on either side of the box represent a 95% confidence interval (CI).

High heterogeneity was observed across all models. Visual inspection of funnel plots revealed no signs of publication bias. In the subscales, visual MI vividness showed no significant differences between groups (SMD = 0.76 (95% CI: [−0.01, 1.52]; *I* ^2^ = 75.54%; PI: [−0.78, 2.29]) (Figure 8). In contrast, kinesthetic MI vividness showed a moderate effect size (SMD = 1.06; 95% CI: [0.42, 1.70]), with considerable heterogeneity (*I* ^2^ = 64.29%) and a PI that included the null effect (PI: −0.17, 2.29]) (Figure 9). Sensitivity analysis confirmed the general robustness of the effect, with values ranging from 0.92 to 1.15 across exclusions (Supporting Information 3).

##### 3.5.2.1. Evidence Synthesis

Despite the presence of high heterogeneity, the consistent directionality of effects and the inclusion of at least four studies with combined quality scores ≥ 7/8 support a classification of moderate evidence. Specifically, individuals with chronic pain exhibited reduced imagery vividness compared to asymptomatic controls.

#### 3.5.3. Mental Chronometry in MI

**FIGURE 10:**
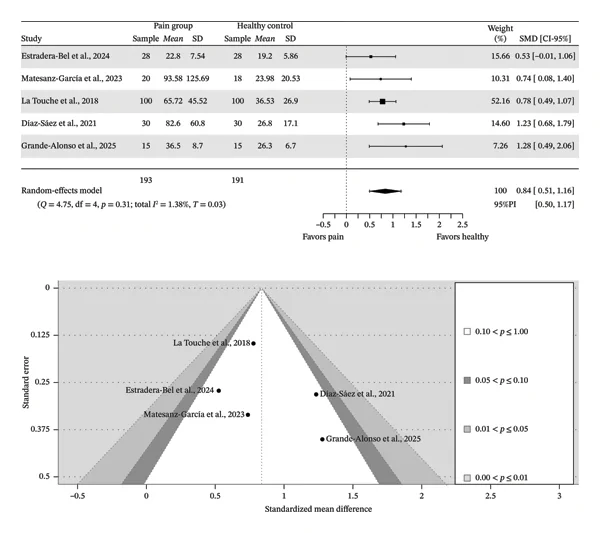
Synthesis forest and funnel plots of global mental chronometry. The forest plot summarizes the results of the included studies (sample size, standardized mean differences [SMDs], and weight). The small boxes with the squares represent the point estimate of the effect size and sample size. The lines on either side of the box represent a 95% confidence interval (CI).

Five studies assessed global mental chronometry performance. The overall analysis showed a significant and moderate effect size (SMD = 0.84; 95% CI: [0.51, 1.16]), with very low heterogeneity (*I* ^2^ = 1.38%) and a narrow PI [0.5, 1.17] (Figure 10). Sensitivity analysis showed stable results across exclusions (SMD range: 0.81–0.92) (Supporting Information 3). Visual inspection of funnel plots indicated no evidence of publication bias.

**FIGURE 11:**
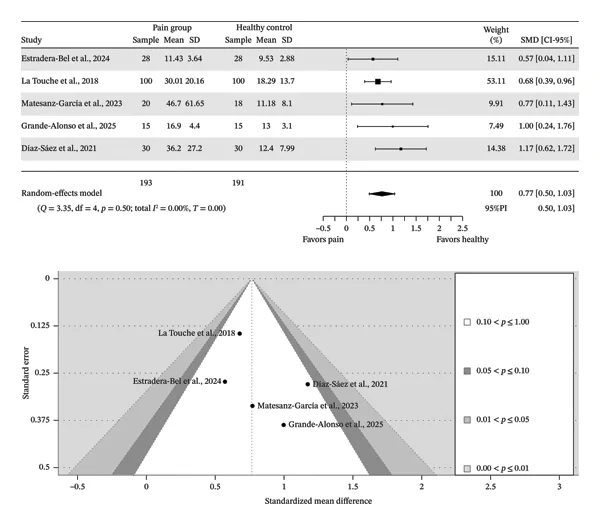
Synthesis forest and funnel plots of visual mental chronometry. The forest plot summarizes the results of the included studies (sample size, standardized mean differences [SMDs], and weight). The small boxes with the squares represent the point estimate of the effect size and sample size. The lines on either side of the box represent a 95% confidence interval (CI).

**FIGURE 12:**
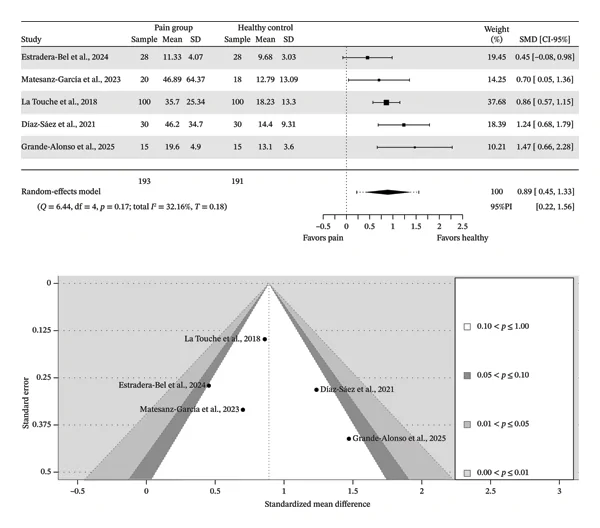
Synthesis forest and funnel plots of kinesthetic mental chronometry. The forest plot summarizes the results of the included studies (sample size, standardized mean differences [SMDs], and weight). The small boxes with the squares represent the point estimate of the effect size and sample size. The lines on either side of the box represent a 95% confidence interval (CI).

Regarding the modalities analysis, visual mental chronometry demonstrated a significant and moderate effect size (SMD = 0.77; 95% CI: [0.50, 1.03], with no observed heterogeneity (*I* ^2^ = 0.0%) and a narrow PI [0.50, 1.03] (Figure 11). Similarly, kinesthetic mental chronometry yielded a comparable result (SMD = 0.89; 95% CI: [0.45, 1.33]), with low heterogeneity (*I* ^2^ = 32.16%) and a PI of [0.22, 1.56] (Figure 12).

##### 3.5.3.1. Evidence Synthesis

Given the low heterogeneity, consistency of effect sizes across subscales, and the high methodological quality of at least four of the five studies, this outcome qualifies as strong evidence. Specifically, individuals with chronic pain exhibited significantly slower performance in mental chronometry tasks compared to asymptomatic controls.

#### 3.5.4. Correlations Between MI Processes and Psychological, Sensory, and Functional Variables

This section specifically describes the correlations between explicit MI processes (ability, vividness, and mental chronometry) and psychological (anxiety, catastrophizing, fear of movement, self‐efficacy [CPSS]), sensory (pain type, sensory disturbances), and functional variables in patients with chronic musculoskeletal pain (Supporting Information 4). Relevant correlation and regression findings from selected studies are summarized below.

Scandola et al. [41] showed that chronic postlesional pain (including neuropathic pain) is associated with greater MI deficits—especially kinesthetic and internal visual—in the painful body regions. In their ANCOVA models, the pain × time effect was significant for internal visual MI (EVI), *F*(1, 26) = 4.91, *p* = 0.036, *η* ^2^ = 0.16 (*r* ≈ 0.40), and for kinesthetic MI (KIN), *F*(1, 26) = 6.41, *p* = 0.018, *η* ^2^ = 0.20 (*r* ≈ 0.45); moreover, KIN decreased over time in the presence of musculoskeletal pain, *F*(1, 18) = 14.97, *p* = 0.001, *η* ^2^ = 0.45 (*r* ≈ 0.67). In patients with carpal tunnel syndrome, Matesanz‐García et al. [44] reported significant negative correlations between kinesthetic imagery ability and both functional impairment (*r* = −0.569; *p* = 0.021) and temporal summation of pain (*r* = −0.515; *p* < 0.05). La Touche et al. [45] showed that fear of movement and pain‐related self‐efficacy explained 16.2% of the variance in visual imagery ability, whereas pain‐related disability and self‐efficacy explained 17.8% of the variance in kinesthetic imagery ability in patients with chronic low back pain.

Alikaj et al. [46] reported that internal visual imagery scores had a significant negative association with maximum perceived pain (standardized *β* = −0.34, *p* < 0.05) and a significant positive association with Pain Catastrophizing Scale (PCS) scores (standardized *β* = 0.66, *p* < 0.001) in patients with familial Mediterranean fever. Grande‐Alonso et al. [49] identified a moderate, significant positive correlation between pain‐related self‐efficacy and kinesthetic MI ability (*r* = 0.54, *p* = 0.035). Regression analysis indicated that pain‐related self‐efficacy accounted for 24.5% of the variance in kinesthetic imagery ability, suggesting that higher self‐efficacy in pain management is linked to enhanced kinesthetic MI performance in patients with chronic shoulder pain.

Finally, Ozcan and Karaali Kul [32] reported significant negative correlations between perceived disability (measured by the Neck Disability Index [NDI]) and all three imagery modalities: internal visual imagery (rs = −0.255, *p* = 0.020), external visual imagery (rs = −0.302, *p* = 0.005), and kinesthetic imagery (rs = −0.324, *p* = 0.003). Negative correlations were also observed between kinesiophobia (assessed with the Tampa Scale for Kinesiophobia [TSK]) and both internal visual imagery (rs = −0.371, *p* = 0.001) and external visual imagery (rs = −0.284, *p* = 0.009). Multiple linear regression analysis showed that perceived disability explained 18.4% of the variance in kinesthetic imagery (*R* ^2^ = 0.184, *p* < 0.001) and 14.9% in external visual imagery (*R* ^2^ = 0.149, *p* < 0.001), while kinesiophobia and pain duration together accounted for 17% of the variance in internal visual imagery (*R* ^2^ = 0.170, *p* = 0.001).

##### 3.5.4.1. Evidence Synthesis

In summary, moderate evidence supports consistent associations across high‐quality studies between perceived disability and reduced MI performance, identifying perceived disability as a key determinant of MI deficits. Moderate evidence also suggests relevant relationships between self‐efficacy, kinesiophobia, and imagery abilities. Finally, limited evidence—due to fewer available studies—indicates that neuropathic pain and functional deficits negatively impact specific modalities of motor imagery, warranting further investigation.

## 4. Discussion

The findings of this meta‐analysis indicate meaningful differences in explicit MI performance between individuals with chronic pain and related pain conditions and healthy controls, particularly across the domains of imagery ability, vividness, and mental chronometry. However, the magnitude and consistency of these effects varied across outcomes and clinical conditions, and therefore should be interpreted in light of the substantial heterogeneity observed in several analyses.

First, overall MI ability was found to be moderately impaired in individuals with chronic pain and related pain populations. This variability is likely attributable to heterogeneity in the pain conditions included, differences in the assessment tools used (e.g., MIQ‐RS, MIQ‐3), and clinical or psychological variability across samples. While some studies reported clear deficits, others found no significant differences, particularly when potentially relevant factors such as pain duration, disability, or emotional distress were not adequately controlled. In this regard, the present findings are broadly consistent with previous observations in implicit MI, particularly in laterality judgment tasks involving painful or movement‐relevant body regions [12, 15]. However, the current review extends this literature by focusing specifically on explicit MI and by differentiating between imagery ability, vividness, and mental chronometry.

A key finding of this review is the substantial heterogeneity observed in several pooled analyses, particularly for imagery ability and vividness. This heterogeneity is likely multifactorial. First, the included populations were clinically diverse, encompassing musculoskeletal, neuropathic, nociplastic, postlesional, and recurrent pain‐related conditions. Second, studies varied in important sample characteristics, including the anatomical region affected, pain duration, disability levels, and psychological features such as kinesiophobia, catastrophizing, and self‐efficacy, all of which may modulate MI performance. Third, methodological variability across studies—including differences in imagery instruments, scoring systems, and chronometry procedures—likely contributed to between‐study inconsistency. These factors may help explain why some studies reported marked deficits, whereas others, such as Cohen‐Aknine et al. [33], found preserved explicit MI performance in specific pain populations.

Regarding imagery vividness, the results suggest a less uniform pattern than that observed for mental chronometry. Although large effects were observed in some vividness‐related analyses, the corresponding PIs included the null effect, indicating uncertainty in the expected effect of future studies. This discrepancy may reflect considerable methodological and clinical heterogeneity, including differences in the assessment of internal versus external visual imagery, differences in the subjective somatic experience required for kinesthetic imagery, and variation in the pain populations studied. Therefore, although reduced vividness may be present in some pain populations, this domain appears less stable and more context‐dependent than mental chronometry.

In contrast, mental chronometry emerged as the most consistent and homogeneous domain across studies. Significant effects were observed in both visual and kinesthetic subscales, as well as in overall chronometry measures. These findings suggest that individuals with chronic pain tend to require more time to generate or simulate imagined movement, supporting the interpretation of altered temporal processing in motor simulation. This pattern is consistent with previous reports of slower performance in motor representation tasks involving painful or movement‐relevant body regions [15]. Compared with the other explicit MI domains, mental chronometry appears to be the most robustly altered dimension in the current evidence base.

The findings concerning kinesthetic imagery are also clinically relevant. Moderate deficits in kinesthetic imagery ability were observed, although these findings were accompanied by high heterogeneity and broad PIs. This suggests that kinesthetic MI may be particularly sensitive to between‐study variability, including differences in pain phenotype, disability, movement‐related fear, and body awareness disturbances. In this sense, kinesthetic imagery may be more vulnerable in some subgroups than in others, but the currently available evidence does not support a uniform impairment across all pain conditions.

The present findings should also be interpreted in relation to previous work on MI and body representation in pain populations. In particular, Breckenridge et al. reported altered left/right judgment task performance in some chronic musculoskeletal pain conditions, supporting the broader idea that pain may be associated with disturbed movement‐related representations [12]. However, the present review extends that the literature by focusing specifically on explicit MI and by distinguishing between imagery ability, vividness, and mental chronometry, which appear to show partially different patterns across studies. Broadly, recent work has emphasized that pain and body representations may influence each other bidirectionally, suggesting that altered MI in pain populations may reflect a wider disturbance in body‐related processing rather than an isolated deficit [50].

Although several neuroimaging and neurophysiological studies have proposed mechanisms that may be compatible with altered MI performance in chronic pain, the present review did not directly assess neural markers. Therefore, mechanistic interpretations should be considered provisional and secondary to the behavioral findings synthesized here. From the perspective of the present meta‐analysis, the most robust conclusion is behavioral: Explicit MI performance, particularly mental chronometry and some kinesthetic dimensions, appears to be altered in pain populations relative to healthy controls.

Taken together, the current findings suggest that explicit MI impairments in pain populations are not uniform across all domains. Rather, they appear to follow a differentiated pattern, with mental chronometry showing the most consistent alteration, imagery ability showing moderate but heterogeneous impairment, and vividness showing the greatest variability. This pattern has important implications for both assessment and interpretation, as it suggests that different explicit MI components may not be equally disrupted across pain conditions and may therefore require different methodological and clinical approaches.

## 5. Limitation and Future Research

This meta‐analysis provides evidence of altered explicit MI performance in chronic pain and related pain populations, yet several limitations should be considered when interpreting the findings and planning future research.

First, there was substantial heterogeneity in pain conditions, duration, intensity, and affected body regions. Although this increases ecological validity, it limits the ability to isolate condition‐specific patterns. The lack of moderator analyses—due to the limited number of available studies and the need to maintain a focused review scope—precluded a deeper exploration of between‐study variability. Future meta‐analyses should consider subgroup analyses based on pain phenotype, pain location, or clinical presentation where sufficient studies are available.

Second, the use of diverse assessment tools (e.g., MIQ‐R, MIQ‐3, VMIQ‐2, TMIQ, and custom chronometry tasks) complicates direct comparisons across studies, particularly because these instruments differ in scoring direction, task demands, and imagery instructions. Greater standardization of explicit MI assessment protocols, including predefined instructions, practice trials, and harmonized scoring procedures, would improve comparability and internal validity.

Third, relatively few studies controlled for psychological and functional factors known to influence MI performance, such as depression, anxiety, pain catastrophizing, kinesiophobia, self‐efficacy, or body awareness disturbances [50, 51]. This limits the interpretability of some between‐group differences and makes it difficult to determine whether the observed impairments are directly related to pain, indirectly related to its psychosocial consequences, or both. Recent work further suggests that mental imagery in chronic pain may be influenced by pain‐related psychological factors and fear‐related responses, reinforcing the need for future studies to incorporate these dimensions more systematically [52, 53].

Fourth, the predominance of cross‐sectional designs restricts causal inference. It remains unclear whether altered explicit MI contributes to pain persistence, emerges as a consequence of long‐standing pain, or reflects a bidirectional relationship. Longitudinal and interventional studies are needed to clarify the temporal and clinical significance of these impairments.

Fifth, although this meta‐analysis focused specifically on explicit MI, the operational distinction between explicit and implicit MI is not always fully clear in the literature. Some vividness or chronometry tasks may recruit additional processes that are not purely explicit. Future studies should provide clearer operational definitions of explicit MI subcomponents and more explicitly distinguish between ability, vividness, and chronometry.

Sixth, one study contributed more than one comparison using a shared control group, which may affect the assumption of independence. Although multilevel models were used to partially address this issue, some influence on standard error estimation cannot be excluded. In addition, means and standard deviations had to be estimated in two studies [32, 33], which may have affected precision.

Finally, small sample sizes in many of the included studies may limit statistical power and reduce the feasibility of more detailed subgroup analyses. Future collaborative and multicenter research using harmonized protocols may help generate larger, more representative datasets and support more refined analyses across pain conditions.

Taken together, these limitations highlight the need for future studies that (1) differentiate explicit MI dimensions more precisely, (2) stratify samples based on clinical and psychological features, (3) use more standardized protocols, and (4) examine the longitudinal and therapeutic relevance of MI‐related impairments across different pain populations.

## 6. Clinical Implications

The results of this meta‐analysis have several relevant clinical implications for the assessment of individuals with chronic pain and related clinical pain conditions. In particular, the findings suggest that explicit MI should not be considered as a unitary construct in clinical settings because different dimensions—especially mental chronometry, imagery ability, and vividness—appear to show different levels of impairment and consistency across studies.

First, the assessment of explicit MI may provide useful complementary information about sensorimotor dysfunction that is not always captured by overt movement evaluations alone. In particular, the relative consistency of the findings in mental chronometry suggests that temporal aspects of imagined movement may be a sensitive domain for identifying pain‐related disturbances in motor simulation. From a clinical perspective, this may support the inclusion of chronometry‐based tasks within broader sensorimotor assessment protocols.

Second, the heterogeneity observed across imagery ability and vividness outcomes suggests that MI‐based assessment should be individualized rather than interpreted in a uniform way. Factors such as pain location, disability level, kinesiophobia, catastrophizing, and self‐efficacy may influence explicit MI performance and may therefore be relevant when selecting the most appropriate modality, perspective, or interpretation of task performance.

Finally, the greater consistency observed in mental chronometry compared with other explicit MI outcomes may have practical implications for prioritizing assessment targets in both clinical and research settings. Identifying which MI dimensions are altered in a given patient may help refine clinical reasoning and may contribute to a more targeted interpretation of movement‐related representational deficits.

## 7. Conclusion

This systematic review and meta‐analysis suggest that explicit MI performance is altered in chronic pain and related pain populations compared with healthy controls. Moderate to large deficits were observed across imagery ability, vividness, and mental chronometry, although the consistency of these findings varied across domains. In particular, substantial heterogeneity was identified in imagery ability and vividness outcomes, reflecting both methodological and clinical variability across the literature. In contrast, mental chronometry showed a more consistent and homogeneous pattern, suggesting that this may be the most robustly altered explicit MI domain in current evidence. Future studies should use more standardized protocols, clearer operational definitions, and more clinically stratified designs to clarify the magnitude and specificity of explicit MI impairments across different pain populations.

## Author Contributions

Roy La Touche: conceptualization, data curation, investigation, methodology, project administration, resources, supervision, validation, visualization, roles/writing–original draft, and writing–review and editing. Ferran Cuenca‐Martínez: conceptualization, investigation, resources, roles/writing–original draft, and writing–review and editing. Jose Vicente León‐Hernández: investigation, roles/writing–original draft, and writing–review and editing. Alba Paris‐Alemany: conceptualization, project administration, supervision, validation, roles/writing–original draft, and writing–review and editing. Miguel A. Sorrel: investigation, roles/writing–original draft, and writing–review and editing. Álvaro Reina‐Varona: data curation, formal analysis, investigation, methodology, software, visualization, roles/writing–original draft, and writing–review and editing.

## Disclosure

This research received no specific grant from any funding agency in the public, commercial, or not‐for‐profit sectors. The work was conducted as part of the authors’ institutional roles at Centro Superior de Estudios Universitarios La Salle.

## Conflicts of Interest

The authors declare no conflicts of interest.

## Supporting Information

Additional supporting information can be found online in the Supporting Information section.

## Supporting information


**Supporting Information 1** Supporting Information 1 shows the results of the quality assessment with the JBI tool for each study.

**Supporting Information 2** Supporting Information 2 shows the results of the quality assessment with the NOS scale for each study.

**Supporting Information 3** Supporting Information 3 presents the results for each sensitivity analysis of the different outcomes, obtained using the leave‐one‐out function.

**Supporting Information 4** Supporting Information 4 summarizes the main correlations and regression coefficients obtained in each study for the psychological variables.

**Supporting Information 5** Supporting Information 5 PRISMA_2020_checklist MI SR.

## Data Availability

The datasets generated and/or analyzed during the current study are available from the corresponding author on reasonable request.
