# Supplementary material for: Explicit Motor Imagery Deficits in Individuals With Chronic Pain: A Systematic Review and Meta‐Analysis
Source: Pain Res Manag. 2026 Sep 7;2026:5805413. doi: 10.1155/prm/5805413 (PMC13548408; doi:10.1155/prm/5805413)
Supplement: Supplementary file 1 [file PRM-2026-5805413-s001.docx]

# **Supplementary Material 1.** Quality Assessment using Johanna-Briggs Institute (JBI) Checklist

| **Study** | **1. Inclusion criteria** | **2. Subjects and setting** | **3. Exposure validity and reliability** | **4. Exposure standardization** | **5. Confounding factors identified** | **6. Confounding factors controlled** | **7. Outcome measures validity and reliability** | **8. Statistical analysis** | **Total** |
| --- | --- | --- | --- | --- | --- | --- | --- | --- | --- |
| Scandola et al., 2016 | Yes | Yes | Not applicable | Yes | Yes | Yes | No | Yes | 7/8 |
| La Touche et al., 2019 | Yes | Yes | Not applicable | Yes | Yes | Yes | Yes | Yes | 7/8 |
| Díaz-Sáez et al. (2020) | Yes | Yes | Not applicable | Yes | Yes | Yes | Yes | Yes | 7/8 |
| Alvarado et al., 2021 | Yes | Yes | Not applicable | Yes | Yes | Yes | Yes | Yes | 7/8 |
| Ozcan & Karaali Kul (2022) | Yes | Yes | Not applicable | Yes | No | No | Yes | Yes | 5/8 |
| Scandola et al., 2022 | Yes | Yes | Not applicable | Yes | Yes | No | Yes | Yes | 6/8 |
| Matesanz- Gracía et al., 2023 | Yes | Yes | Not applicable | Yes | Yes | Yes | Yes | Yes | 7/8 |
| Cohen-Aknine et al., 2025 | Yes | Yes | Not applicable | Yes | Yes | No | Yes | Yes | 6/8 |
| Alikaj et al., 2024 | Yes | Yes | Not applicable | Yes | Yes | No | Yes | Yes | 6/8 |
| Estradera-Bel et al., 2024 | Yes | Yes | Not applicable | Yes | Yes | Yes | Yes | Yes | 7/8 |
| Grande-Alonso et al., 2025 | Yes | Yes | Not applicable | Yes | Yes | Yes | Yes | Yes | 7/8 |
| Alaca et al., 2025 | Yes | Yes | Not applicable | Yes | Yes | Yes | Yes | Yes | 7/8 |
