# Supplementary material for: Explicit Motor Imagery Deficits in Individuals With Chronic Pain: A Systematic Review and Meta‐Analysis
Source: Pain Res Manag. 2026 Sep 7;2026:5805413. doi: 10.1155/prm/5805413 (PMC13548408; doi:10.1155/prm/5805413)
Supplement: Supplementary file 2 [file PRM-2026-5805413-s002.docx]

# **Supplementary Material 2.** Quality Assessment using Newcastle-Ottawa Scale (NOS)

| **Study** | **Selection (item nº1)** | **Selection (item nº2)** | **Selection (item nº3)** | **Selection (item nº4)** | **Comparability (factors)** | **Outcome (item nº1)** | **Outcome (item nº2)** | **Total** |
| --- | --- | --- | --- | --- | --- | --- | --- | --- |
| Scandola et al., 2016 | ★ | – | ★ | ★★ | ★★ | ★ | – | 7/9 |
| La Touche et al., 2019 | ★ | – | ★ | ★★ | ★★ | ★ | ★ | 8/9 |
| Díaz-Sáez et al. (2020) | ★ |  | ★ | ★★ | ★★ | ★ | ★ | 8/9 |
| Alvarado et al., 2021 | ★ | – | ★ | ★ | ★★ | ★ | ★ | 7/9 |
| Ozcan & Karaali Kul (2022) | ★ | – | ★ | ★ | - | ★ | ★ | 6/9 |
| Scandola et al., 2022 | ★ | – | ★ | ★★ | ★ | ★ | ★ | 7/9 |
| Matesanz-García et al., 2023 | ★ | – | ★ | ★ | ★★ | ★ | ★ | 7/9 |
| Cohen-Aknine et al., 2025 | ★ | – | ★ | ★★ | ★ | ★ | - | 6/9 |
| Alikaj et al., 2024 | ★ | – | ★ | ★★ | ★ | ★ | ★ | 7/9 |
| Estredera-Bel et al., 2024 | ★ | – | ★ | ★ | ★★ | ★ | ★ | 7/9 |
| Grande-Alonso et al., 2025 | ★ | – | ★ | - | ★★ | ★ | ★ | 7/8 |
| Alaca et al., 2025 | ★ | – | ★ | ★★ | ★★ | ★ | ★ | 8/9 |
