# Supplementary material for: Explicit Motor Imagery Deficits in Individuals With Chronic Pain: A Systematic Review and Meta‐Analysis
Source: Pain Res Manag. 2026 Sep 7;2026:5805413. doi: 10.1155/prm/5805413 (PMC13548408; doi:10.1155/prm/5805413)
Supplement: Supplementary file 3 [file PRM-2026-5805413-s003.docx]

**Supplementary Material 3.** Leave-One-Out Sensitivity Analyses for Meta-Analytic Robustness

**1. Leave-One-Out for total chronometry**

| Omitted Study | Estimate | SE | t-value | p-value | CI.lb | CI.ub | Q | Qp | tau^2 | I^2 | H^2 |
| --- | --- | --- | --- | --- | --- | --- | --- | --- | --- | --- | --- |
| Estradera-Bel et al., 2024 | 0.9194 | 0.1290 | 7.1251 | 0.0057 | 0.5088 | 1.3301 | 3.2158 | 0.3595 | 0.0142 | 16.3542 | 1.1955 |
| Díaz-Sáez et al., 2020 | 0.7690 | 0.1038 | 7.4087 | 0.0051 | 0.4387 | 1.0993 | 2.4275 | 0.4885 | 0.0000 | 0.0077 | 1.0001 |
| Matesanz-García et al., 2023 | 0.8788 | 0.1615 | 5.4420 | 0.0122 | 0.3649 | 1.3928 | 4.6582 | 0.1986 | 0.0309 | 32.4600 | 1.4806 |
| La Touche et al., 2019 | 0.9135 | 0.1878 | 4.8642 | 0.0166 | 0.3158 | 1.5112 | 4.4199 | 0.2195 | 0.0520 | 34.2895 | 1.5218 |
| Grande-Alonso et al., 2025 | 0.8019 | 0.1186 | 6.7587 | 0.0066 | 0.4243 | 1.1794 | 3.4412 | 0.3285 | 0.0000 | 0.0044 | 1.0000 |

**2. Leave-One-Out for vividness for external visual imagery**

| Omitted Study | Estimate | SE | t-value | p-value | CI.lb | CI.ub | Q | Qp | tau^2 | I^2 | H^2 |
| --- | --- | --- | --- | --- | --- | --- | --- | --- | --- | --- | --- |
| Scandola et al., 2022 | 0.6301 | 0.2776 | 2.2698 | 0.1513 | -0.5643 | 1.8244 | 9.4870 | 0.0087 | 0.1831 | 78.0206 | 4.5497 |
| Alaca et al., 2025 | 0.6138 | 0.2778 | 2.2096 | 0.1577 | -0.5814 | 1.8089 | 7.3613 | 0.0252 | 0.1670 | 73.6039 | 3.7884 |
| Scandola et al., 2016 | 0.8451 | 0.3140 | 2.6915 | 0.1148 | -0.5059 | 2.1961 | 11.7765 | 0.0028 | 0.2463 | 81.7174 | 5.4697 |
| Alvarado et al., 2021 | 0.9455 | 0.2249 | 4.2043 | 0.0522 | -0.0221 | 1.9131 | 4.8163 | 0.0900 | 0.0887 | 58.4456 | 2.4065 |

**3. Leave-One-Out for chronometry for external visual imagery**

| Omitted Study | Estimate | SE | t-value | p-value | CI.lb | CI.ub | Q | Qp | tau^2 | I^2 | H^2 |
| --- | --- | --- | --- | --- | --- | --- | --- | --- | --- | --- | --- |
| Estradera-Bel et al., 2024 | 0.8278 | 0.1153 | 7.1778 | 0.0056 | 0.4608 | 1.1948 | 2.7472 | 0.4323 | 0.0135 | 15.9525 | 1.1898 |
| Díaz-Sáez et al., 2020 | 0.6973 | 0.0620 | 11.2399 | 0.0015 | 0.4999 | 0.8948 | 0.8803 | 0.8302 | 0.0000 | 0.0000 | 1.0000 |
| Matesanz-García et al., 2023 | 0.7778 | 0.1240 | 6.2721 | 0.0082 | 0.3831 | 1.1724 | 3.3480 | 0.3410 | 0.0079 | 11.1272 | 1.1252 |
| La Touche et al., 2019 | 0.8659 | 0.1426 | 6.0703 | 0.0090 | 0.4119 | 1.3198 | 2.5645 | 0.4637 | 0.0044 | 4.2685 | 1.0446 |
| Grande-Alonso et al., 2025 | 0.7468 | 0.1095 | 6.8210 | 0.0064 | 0.3984 | 1.0953 | 2.9621 | 0.3975 | 0.0000 | 0.0000 | 1.0000 |

**4. Leave-One-Out for vividness for kinesthetic imagery**

| Omitted Study | Estimate | SE | t-value | p-value | CI.lb | CI.ub | Q | Qp | tau^2 | I^2 | H^2 |
| --- | --- | --- | --- | --- | --- | --- | --- | --- | --- | --- | --- |
| Scandola et al., 2022 | 1.0704 | 0.2810 | 3.8093 | 0.0625 | -0.1386 | 2.2795 | 8.6001 | 0.0136 | 0.1818 | 76.4780 | 4.2513 |
| Alaca et al., 2025 | 0.8474 | 0.0820 | 10.3353 | 0.0092 | 0.4946 | 1.2002 | 0.6746 | 0.7137 | 0.0000 | 0.0000 | 1.0000 |
| Scandola et al., 2016 | 1.1261 | 0.2664 | 4.2274 | 0.0517 | -0.0200 | 2.2722 | 7.8591 | 0.0197 | 0.1601 | 73.3654 | 3.7545 |
| Alvarado et al., 2021 | 1.1795 | 0.2396 | 4.9218 | 0.0389 | 0.1484 | 2.2106 | 5.4582 | 0.0653 | 0.1111 | 62.6570 | 2.6779 |

**5. Leave-One-Out for chronometry for kinesthetic imagery**

| Omitted Study | Estimate | SE | t-value | p-value | CI.lb | CI.ub | Q | Qp | tau^2 | I^2 | H^2 |
| --- | --- | --- | --- | --- | --- | --- | --- | --- | --- | --- | --- |
| Estradera-Bel et al., 2024 | 0.9679 | 0.1346 | 7.1900 | 0.0055 | 0.5395 | 1.3963 | 3.5508 | 0.3142 | 0.0073 | 9.0850 | 1.0999 |
| Díaz-Sáez et al., 2020 | 0.8118 | 0.1530 | 5.3077 | 0.0131 | 0.3251 | 1.2986 | 4.5326 | 0.2094 | 0.0078 | 9.8087 | 1.1088 |
| Matesanz-García et al., 2023 | 0.9386 | 0.2016 | 4.6559 | 0.0187 | 0.2970 | 1.5801 | 6.1353 | 0.1052 | 0.0730 | 52.9155 | 2.1238 |
| La Touche et al., 2019 | 0.9260 | 0.2311 | 4.0065 | 0.0279 | 0.1905 | 1.6615 | 6.4151 | 0.0931 | 0.1122 | 52.7790 | 2.1177 |
| Grande-Alonso et al., 2025 | 0.8272 | 0.1428 | 5.7946 | 0.0102 | 0.3729 | 1.2815 | 4.1956 | 0.2411 | 0.0141 | 19.0194 | 1.2349 |
