# Supplementary material for: Explicit Motor Imagery Deficits in Individuals With Chronic Pain: A Systematic Review and Meta‐Analysis
Source: Pain Res Manag. 2026 Sep 7;2026:5805413. doi: 10.1155/prm/5805413 (PMC13548408; doi:10.1155/prm/5805413)
Supplement: Supplementary file 4 [file PRM-2026-5805413-s004.docx]

**Supplementary Material 4.** Summary Table of Correlations and Regressions.

| **Author(s)** | **Psychological/Sensory Variables** | **Statistical analysis and mathematical data** |
| --- | --- | --- |
| Scandola et al. (2017) | Chronic post-lesional pain (including neuropathic pain) | Greater deficits in kinesthetic and internal visual motor imagery for body areas affected by pain (p<0.05) |
| Matesanz-García et al. (2023) | Functional deficit, temporal summation | r=−0.569 (functional deficit, p=0.021), r=−0.515 (temporal summation, p<0.05) |
| La Touche et al. (2019) | Kinesiophobia, self-efficacy (CPSS), pain-related disability | Visual: Kinesiophobia and CPSS 16.2% variance; Kinesthetic: disability and CPSS 17.8% variance |
| Alikaj et al. (2024) | Maximum perceived pain, catastrophizing | IVI: β=−0.34 (max pain, p<0.05), β=0.66 (PCS, p<0.001) |
| Grande-Alonso et al. (2025) | Self-efficacy (CPSS) | r=0.54 with kinesthetic ability (p=0.035), CPSS explains 24.5% variance in kinesthetic ability |
| Ozcan and Karaali Kul (2022) | Perceived disability, kinesiophobia, pain duration | IVI: R²=0.170 (kinesiophobia, pain duration); EVI: R²=0.149 (disability); KI: R²=0.184 (disability, affective pain intensity) |
